# *Albino Leaf 2* is involved in the splicing of chloroplast group I and II introns in rice

**DOI:** 10.1093/jxb/erw296

**Published:** 2016-08-19

**Authors:** Changhong Liu, Haitao Zhu, Yi Xing, Jianjie Tan, Xionghui Chen, Jianjun Zhang, Haifeng Peng, Qingjun Xie, Zemin Zhang

**Affiliations:** State Key Laboratory for Conservation and Utilization of Subtropical Agro-Bioresources, Guangdong Provincial Key Laboratory of Plant Molecular Breeding, College of Agriculture, South China Agricultural University, Guangzhou 510642, China

**Keywords:** Albino leaf, chloroplast development, CRS1, group I intron, group II intron, rice, RNA splicing.

## Abstract

Chloroplasts play an essential role in plant growth and development through manipulating photosynthesis and the production of hormones and metabolites. Although many genes or regulators involved in chloroplast biogenesis and development have been isolated and characterized, identification of novel components is still lacking. We isolated a rice (*Oryza sativa*) mutant, termed *albino leaf 2* (*al2*), using genetic screening. Phenotypic analysis revealed that the *al2* mutation caused obvious albino leaves at the early developmental stage, eventually leading to *al2* seedling death. Electron microscopy investigations indicated that the chloroplast structure was disrupted in the *al2* mutants at an early developmental stage and subsequently resulted in the breakdown of the entire chloroplast. Molecular cloning illustrated that *AL2* encodes a chloroplast group IIA intron splicing facilitator (CRS1) in rice, which was confirmed by a genetic complementation experiment. Moreover, our results demonstrated that *AL2* was constitutively expressed in various tissues, including green and non-green tissues. Interestingly, we found that the expression levels of a subset of chloroplast genes that contain group IIA and IIB introns were significantly reduced in the *al2* mutant compared to that in the wild type, suggesting that *AL2* is a functional CRS1 in rice. Differing from the orthologous CRS1 in maize and Arabidopsis that only regulates splicing of the chloroplast group II intron, our results demonstrated that the *AL2* gene is also likely to be involved in the splicing of the chloroplast group I intron. They also showed that disruption of *AL2* results in the altered expression of chloroplast-associated genes, including chlorophyll biosynthetic genes, plastid-encoded polymerases and nuclear-encoded chloroplast genes. Taken together, these findings shed new light on the function of nuclear-encoded chloroplast group I and II intron splicing factors in rice.

## Highlight

The *Albino Leaf 2* (*AL2*) gene is involved in the splicing of chloroplast group I and II introns and is responsible for chloroplast development in rice.

## Introduction

Chloroplasts are essential for plant development and growth, through manipulating the fixation of CO_2_ and biosynthesis of carbon skeletons and other physiological processes (Sakamoto *et al.*, 2008; Jarvis and Lopez-Juez, 2013). Accumulating evidence has shown the importance of chloroplast biogenesis and development during germination for plant vitality, seed set and growth (Lopez-Juez and Pyke, 2005; Pogson and Albrecht, 2011). Chloroplast biogenesis is initiated from proplastids through endosymbiosis from an ancestor of extant cyanobacteria and is dependent on the coordinated expression of genes encoded in both nuclear and plastid genomes (Lopez-Juez and Pyke, 2005; Sakamoto *et al.*, 2008; Kessler and Schnell, 2009). The development of chloroplasts differs between organs and species depending on the specialization of tissues and stage of development. For example, distinct phenotypes between cotyledons and true leaves were observed in *variegated* (*var*) and *snowy cotyledon* (*sco*) mutants in Arabidopsis, respectively (Albrecht *et al.*, 2006, 2008, 2010; Liu *et al.*, 2010), i.e. chlorotic true leaves but green cotyledons in the *var* and chlorotic or bleached cotyledons but green true leaves in the *sco* mutants. It is evident that nucleus-encoded polymerases (NEPs) and plastid-encoded polymerases (PEPs) involved in gene transcription, RNA maturation, protein translation and modification have great effect on the biogenesis of chloroplasts (Hedtke *et al.*, 1997; Pogson and Albrecht, 2011; Yu *et al.*, 2014). Recent studies indicated that a large group of nuclear-encoded pentatricopeptide repeats proteins (PPRs) involved in RNA processing, splicing, editing, stability, maturation and translation are critical for chloroplast development (Pogson and Albrecht, 2011). Besides PPRs, a group of chloroplast RNA splicing and ribosome maturation (CRM) domain proteins were also shown to be required for chloroplast development, which regulate the splicing of certain introns in chloroplasts and/or mitochondria (de Longevialle *et al.*, 2010). In general, primary RNA transcripts in chloroplasts are spliced by a group of ribozymes via the same chemical steps as spliceosome-mediated splicing in the nucleus (de Longevialle *et al.*, 2010; Borner *et al.*, 2015). Based on the conserved structures and different splicing mechanisms, the introns of certain chloroplast and mitochondrial genes are classified into two main families in plants, group I and group II. Twenty group II introns and only one group I intron have been identified in the chloroplast genome of Arabidopsis, whereas 17 group II introns and one group I intron have been described in maize and rice (de Longevialle *et al.*, 2010; Bonen and Vogel, 2001).

Splicing of group I introns is mediated through a two-step phosphoryl transfer reaction (Kruger *et al.*, 1982). So far, only the intron of *trnL* has been characterized as a group I intron in chloroplasts and its splicing requires the function of matK, ZmRNC1, ZmWTF1 and AtCFM2 (Asakura and Barkan, 2007; Watkins *et al.*, 2007; Asakura *et al.*, 2008; Kroeger *et al.*, 2009; Zoschke *et al.*, 2010). Group II introns are divided into four subgroups: IIA, IIB, IIC and IID (Michel *et al.*, 1989; Toor *et al.*, 2001). Many group II intron splicing factors have been identified and characterized, including AtnMat2 (Keren *et al.*, 2009), ORGANELLAR TRANSCRIPT processing 43 (OTP43) (de Longevialle *et al.*, 2007; Keren *et al.*, 2012), mCSF1 (Zmudjak *et al.*, 2013), RUG3 (Kuhn *et al.*, 2011) and ABA overly-sensitive 5 (ABO5) (Liu *et al.*, 2010) in mitochondria, and chloroplast RNA splicing 1 (CRS1) (Ostersetzer *et al.*, 2005; Till *et al.*, 2001), ZmRNC1 (Watkins *et al.*, 2007), AtCFM2 (Asakura and Barkan, 2007) and Zm-mTERF4 (Hammani and Barkan, 2014) in chloroplasts. Among these proteins, CRS1 is the first defined CRM protein from maize, which contains three CRM domains (Jenkins *et al.*, 1997; Till *et al.*, 2001). Sixteen and fourteen proteins containing one or more CRM domains are identified in Arabidopsis and rice, respectively. Besides CRS1, four more CRM proteins have been characterized as chloroplast splicing factors: CRM Family Member 2 (CFM2), CFM3, CRS2-associated factors 1 (CAF1) and CAF2 (Barkan *et al.*, 2007; de Longevialle *et al.*, 2010). CRS1 only functions in the splicing of the *atpF* intron (group IIA), whereas the function of maize CFM2 is associated with one group I intron (*trnL* intron) and two group II introns (*ndhA* intron, *ycf3* intron 1), and Arabidopsis CFM2 also promotes the splicing of the additional group II intron (*clpP* intron) (Asakura and Brakan, 2007; Asakura *et al.*, 2008). Differing from other splicing factors in the CRM family, CFM3 is dual-targeted to chloroplasts and mitochondria. In chloroplasts, the function of CFM3 is associated with a subset of group II introns (*ndhB*, *rpl16*, *rps16*, *trnG*, *petB* and *petD* introns) and regulates their splicing in rice (de Longevialle *et al.*, 2010). CSR2 binds to CAF1 and CAF2 to form the complexes CRS2-CAF1 and CRS2-CAF2, respectively, which are required for the splicing of a subset of group IIB introns, including the introns of *ndhA*, *ndhB*, *petB*, *petD*, *rpl16*, *rps16*, *trnG* and *ycf3* in maize and *rpoC1*and *ClpP* in Arabidopsis (Ostheimer *et al.*, 2003; Asakura and Barkan, 2006; de Longevialle *et al.*, 2010). To the best of our knowledge, most of the group II intron splicing factors were identified from Arabidopsis or maize, whereas these factors, particularly the CRM proteins in rice, are either not identified or their functions are not fully characterized.

We describe the function of a nuclear-encoded splicing factor termed *Albino Leaf 2* (*AL2*) that encodes a chloroplast group IIA intron splicing factor CRS1, which is required for chloroplast development in rice. Our results suggest that in contrast to the function of the orthologous CRS1 in maize and Arabidopsis, *AL2* probably participates in the splicing of both chloroplast group I and II introns in rice. In addition, *AL2* appears to coordinate the expression of a subset of chloroplast-associated genes to regulate chloroplast development in rice.

## Materials and methods

### Plant materials and growth conditions

The rice *japonica* cultivar Zhonghua 11 was used as the wild type. The T-DNA insertion library with Zhonghua 11 background was obtained from the Shanghai Institute of Plant Physiology and Ecology, Chinese Academy of Sciences. All rice seeds in this study were propagated in a paddy field in Guangzhou, China. For laboratory work, rice plants were grown in the greenhouse under a 16-h light/8-h dark cycle at 30 ºC with the given light intensity (1000 mmol m^−2^ s^−1^). No significant differences were observed when *albino leaf* (*al2*) mutants were grown in the greenhouse versus the paddy field.

### Electron microscopy

The samples prepared for electron microscopy were fixed overnight at 4 °C with 2.5% glutaraldehyde in 0.1M phosphate buffer (pH 7.4), and then the samples were dehydrated in a graded ethanol series followed by substitution with a graded isoamyl acetate series. The samples were critical point dried, sputter coated with gold and observed using a HITACHI S-3 000N scanning electron microscope at 10kV. For transmission electron microscopy analysis, samples were fixed overnight at 4 °C with 2.5% glutaraldehyde in 0.1M phosphate buffer (pH 7.4). After washing with phosphate buffer three times, samples were fixed overnight in 2% (v/v) OsO4 in phosphate buffer. After staining and dehydration in a grade ethanol series, the samples were submerged with LR White resin and polymerized for 2 d. Ultrathin cross-sections were prepared with a Leica EM UC6 ultra-microtome and observed under a Tecnai Spirit (120kV) transmission electron microscope.

### Analysis of the T-DNA insertion locus in *al2* mutant

Inverse polymerase chain reaction (IPCR) was used to isolate the flanking sequence of T-DNA (Ochman *et al.*, 1988). Nested primers of the T-DNA right border primers were C1 and C2, and those of the left border primers were H1 and H2. The genomic DNA was digested by H*in*dIII. Primers for testing of the T-DNA inserting locus were AL2-11310 and 5TF1 for the left site and AL2-11655 and 5TR1 for the right. Primers sequences are listed in Supplementary Table S1 at *JXB* online.

### Plasmid construct and transformation

To produce the *AL2* complementation transgenic plants, a 6.2kb DNA fragment of *AL2* was amplified from wild type using their corresponding primer pairs listed in Supplementary Table S1. The amplified DNA fragment was confirmed by DNA sequencing and cloned into the binary vector pCAMBIA1301 (Promega, Madison, WI, USA) and then transformed into *al2* heterozygotes. To generate the interfering *AL2* construct, a 549-bp fragment from the specific coding region of *AL2* was amplified from wild-type complementary DNA (cDNA) templates using their corresponding primer pairs listed in Supplementary Table S1. The DNA fragment was confirmed by sequencing and cloned into the binary vector pCAMBIA1301-35S. The final constructs were electroporated into *Agrobacterium tumefaciens* strain EHA105 for rice transformations that were conducted as previously described (Toki *et al.*, 2006).

### Histological β-glucuronidase (GUS) assay

GUS activity analysis was performed following a standard protocol (Jefferson *et al.*, 1987). Transgenic plant tissues were incubated in X-gluc buffer [0.1mol l^−1^ K_2_HPO_4_ (pH 7.0), 0.1mol l^−1^ KH_2_PO_4_ (pH 7.0), 5 mmol l^−1^ K_3_Fe (CN)_6_, 5 mmol l^−1^ K_4_Fe(CN)_6_•3H_2_O, 0.1% Triton X-100, 20% methanol, 1mg ml^−1^ X-Gluc] at 37 °C for 2h and then cleared by ethanol. Stained samples were photographed using a Cannon digital camera and stereoscope (OLYMPUS SZX12) and further sliced using resin sections (Leica HistoResin) and analysed under a light microscope (OLYMPUS BX51).

### RNA extraction and quantitative real-time (qRT)-PCR assay

Total RNA was extracted using the RNeasy Plant Mini kit (Qiagen) according to the manufacturer’s instructions. The first strand of cDNA was synthesized using TransScript First-Strand cDNA Synthesis SuperMix (TransGen Biotech) and qRT-PCR was performed as previously described (Chen *et al.*, 2015). The relative expression level of the target gene was normalized to that of rice gene *actin1*. All primers used in qRT-PCR are listed in Supplementary Table S1.

### Chlorophyll detection

Total chlorophyll was determined in triplicate according to the method described previously (Hartmut, 1983). Extracts were obtained from the sixth leaves or seedlings at different growth stages. Approximately 0.2g of fresh tissue was homogenized in 5ml of 80% acetone for 12h in darkness. Spectrophotometric quantification was carried out in a Gene Quant spectrophotometer (GE Healthcare) and followed with calculations: Chl *a*=12.21×A663−2.81×A646, and Chl *b*=20.13×A646–5.03×A663 (μg ml^−1^).

## Results

### Phenotype of *al2* mutants

**Fig. 1.**
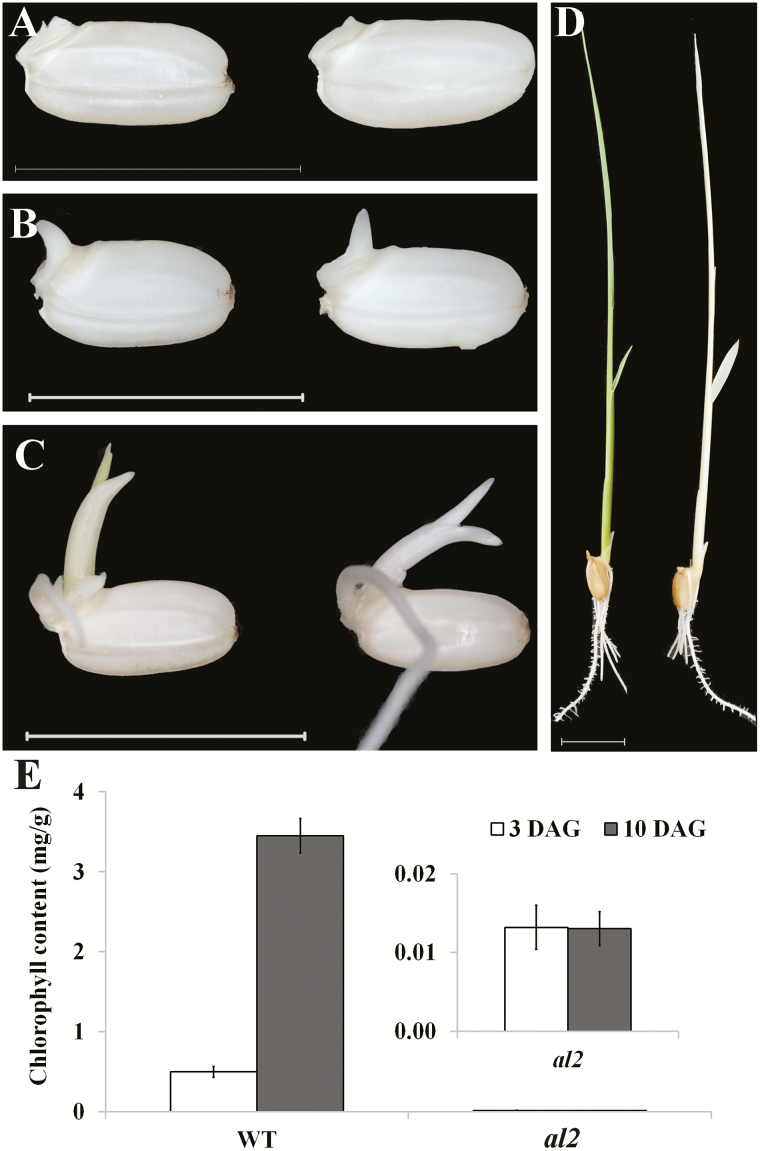
Characterization of *al2*. Phenotype of *al1* during germination at (A) 1 d after germination (DAG), (B) 2 DAG, (C) 4 DAG and (D) 10 DAG. (E) Chlorophyll content of *al2* at 4 and 10 DAG. In each panel, from left to right is wild type and *al2*, respectively. Bar: 1cm (A–C); 2cm (D).

To identify novel genes or regulators involved in chloroplast development, a rice T-DNA insertion population (Zhonghua 11 background) was screened for mutants that exhibited defects in leaf colour, termed *albino leaf* mutants (*als*). Dozens of *als* were obtained through phenotypic investigation. Among these *als*, the *al2* mutant has an interesting and distinct phenotype compared to the others. The *al2* mutant showed no significant difference compared to the wild type at the germination stage (Fig. 1A, B), but it had an apparent albino phenotype in the young buds (Fig. 1C). During seedling growth, the albino phenotype became more obvious and spread around the entire leaf at the third-leaf stage in the *al2* mutant (Fig. 1D), which eventually led to the seedling lethal *al2* mutant. To quantify the changes in this albino phenotype, chlorophyll contents were measured in *al2* mutant and wild type. Consistent with their phenotypes, the chlorophyll contents were significantly reduced in *al2* compared with that of the wild type (Fig. 1E). These data suggested that the albino phenotype of *al2* mutants might be caused by defects in chlorophyll metabolism or the breakdown of entire chloroplasts.

### Chloroplasts are disrupted in *al2* mutants

**Fig. 2.**
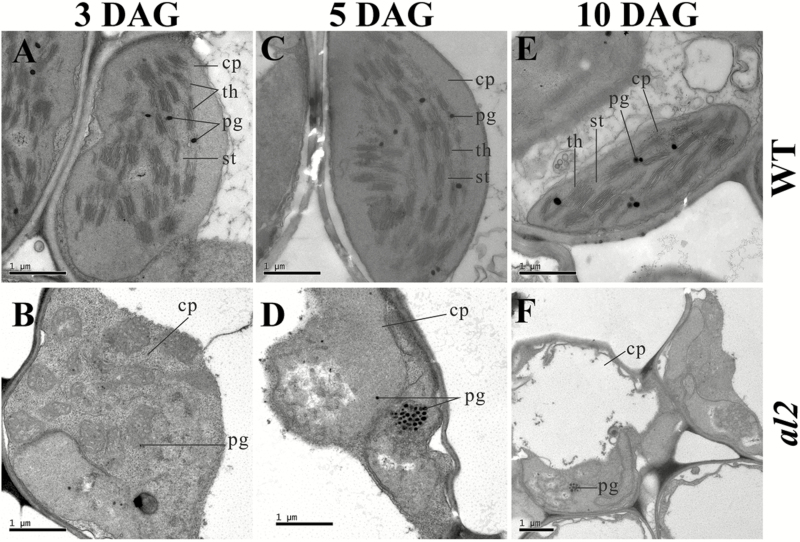
Electron microscopy observation of *al2* leaf. Chloroplasts in wild type mesophyll cells at the (A) 3 DAG, (C) 5 DAG and (E)10 DAG, respectively. Chloroplast in *al2* mutant mesophyll cells at (B) 3 DAG, (D) 5 DAG and (F) 10 DAG, respectively. Chloroplasts of the wild type have abundant and well-ordered thylakoid, whereas those of the *al2* mutant have no similar structures. cp, chloroplast; DAG, day after germination; pg, plastoglobuli; st, stroma; th, thylakoid. Bar, 1 µm.

To further investigate the albino leaf phenotype of *al2*, we observed the chloroplast structure of *al2* mutants and wild type in leaves by electron microscopy. Because the albino phenotype of the *al2* mutant was developed from the germination to the third-leaf stage, leaves of *al2* and wild type from 3 d after germination (DAG), 5 DAG and 10 DAG were selected for microscopy analysis. Our results showed that the development of chloroplasts was significantly affected in *al2* leaves at 3 DAG (Fig. 2A, D), and chloroplasts were severely disrupted in *al2* at 5 DAG, particularly in the formation of thylakoids (Fig. 2B, E). The most apparent defective phenotype of chloroplasts in *al2* was observed at 10 DAG compared to that in wild type (Fig. 2C, F). Taken together, our results indicated that the albino leaf phenotype of the *al2* mutant resulted from the abnormal development of chloroplasts, suggesting that the *AL2* gene is required for chloroplast development in rice.

### Molecular cloning of the *AL2* gene

**Fig. 3.**
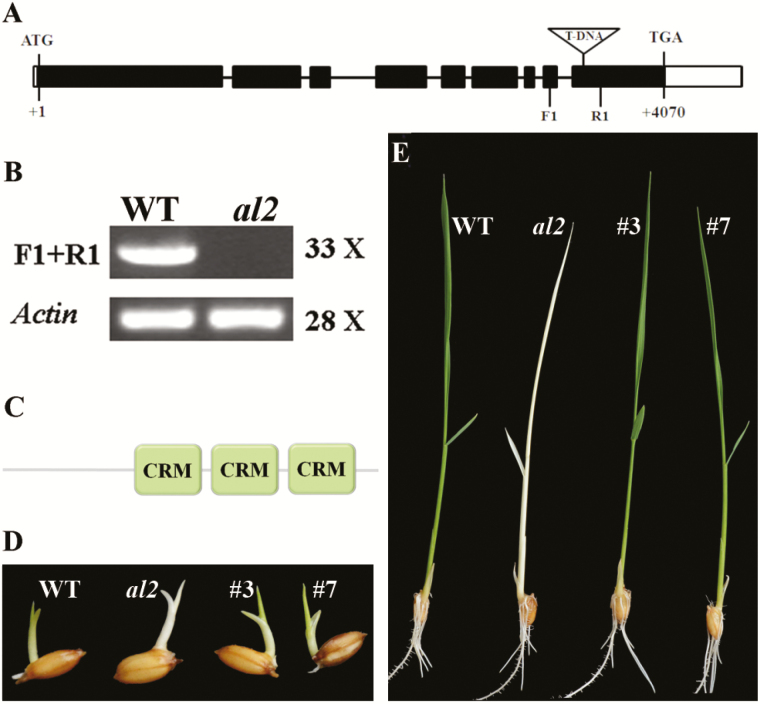
Molecular cloning of *AL2*. (A) Schematic representation of the *AL2* gene. Black boxes represent the exon, the lines represent the intron and the white boxes represent the untranslated region (UTR). The putative star codon (ATG) and stop codon (TGA) are located at the +1 and +4070 positions, respectively. The T-DNA is inserted into the ninth exon of *Os09g19850*. (B) Transcript level of *AL2* in wild type and *al2* as determined by semi-quantitative PCR with the specific primers indicated in panel A. (C) Distribution of three CRM domains in AL2 protein. (D) Germination phenotype of complementation lines. (E) Seedling phenotype of complementation lines. Panels D, E: #3 and #7 are the *al2* complementation lines 3 and 7, respectively. X, PCR cycle.

We backcrossed the *al2* mutant with wild type and did a segregation analysis in the F_2_ population based on the *al2* mutant phenotype. The segregation ratio between *al2* mutant and wild type was ~1:3 (χ^2^=0.120), indicating that *al2* was controlled by a single recessive gene. We then performed inverse PCR and isolated the genomic DNA fragment flanking the T-DNA insertion region in the *al2* mutant. Analysis of the resulting sequences via the BLAST function in the NCBI database revealed that T-DNA was inserted into the ninth exon of the *Os09g19850* gene, which is comprised of nine exons and eight introns (Fig. 3A). Semi-quantitative PCR analysis indicated that the transcript level of *Os09g19850* was completely eliminated in the *al2* mutants (Fig. 3B). These data all suggested that *Os09g19850* is a strong candidate for the *AL2* gene. Gene annotation showed that *Os09g19850* encodes a chloroplast group IIA intron splicing facilitator CRS1 in rice (http://rice.plantbiology.msu.edu) containing three CRS1-YhbY domains (also called the CRM domain) (Fig. 3C). Protein alignment analysis indicated that AL2/CRS1 proteins were highly conserved among various organisms. Moreover, phylogenetic analysis demonstrated that the AL2-like proteins in the plant kingdom were grouped into two clusters: monocot plants and dicot plants (Supplementary Fig. S1).

To verify the identity of *AL2*, a genetic complementation assay was performed. A 6225-bp wild-type genomic DNA fragment of *Os09g19850*, containing the putative promoter (2318bp), the coding sequences (including the intron sequences) and a part of the 3ʹ-untranslated region (246bp), was cloned into a binary vector and transformed into *al2* heterozygous plants due to the lethality of the *al2* homozygous plants. Five independent transgenic lines within the *al2* homozygote background were obtained, and two of them were used as representatives in the following studies. In these two transgenic lines, the albino leaf phenotype of *al2* was fully rescued by the *AL2* transgene (Fig. 3D, E), and the chlorophyll changes (Supplementary Fig. S2). Therefore, we concluded that *Os09g19850* is the *AL2* gene, and the disruption of its function results in the albino leaf phenotype.

### Disruption of *AL2* leads to an *al2*-like phenotype

**Fig. 4.**
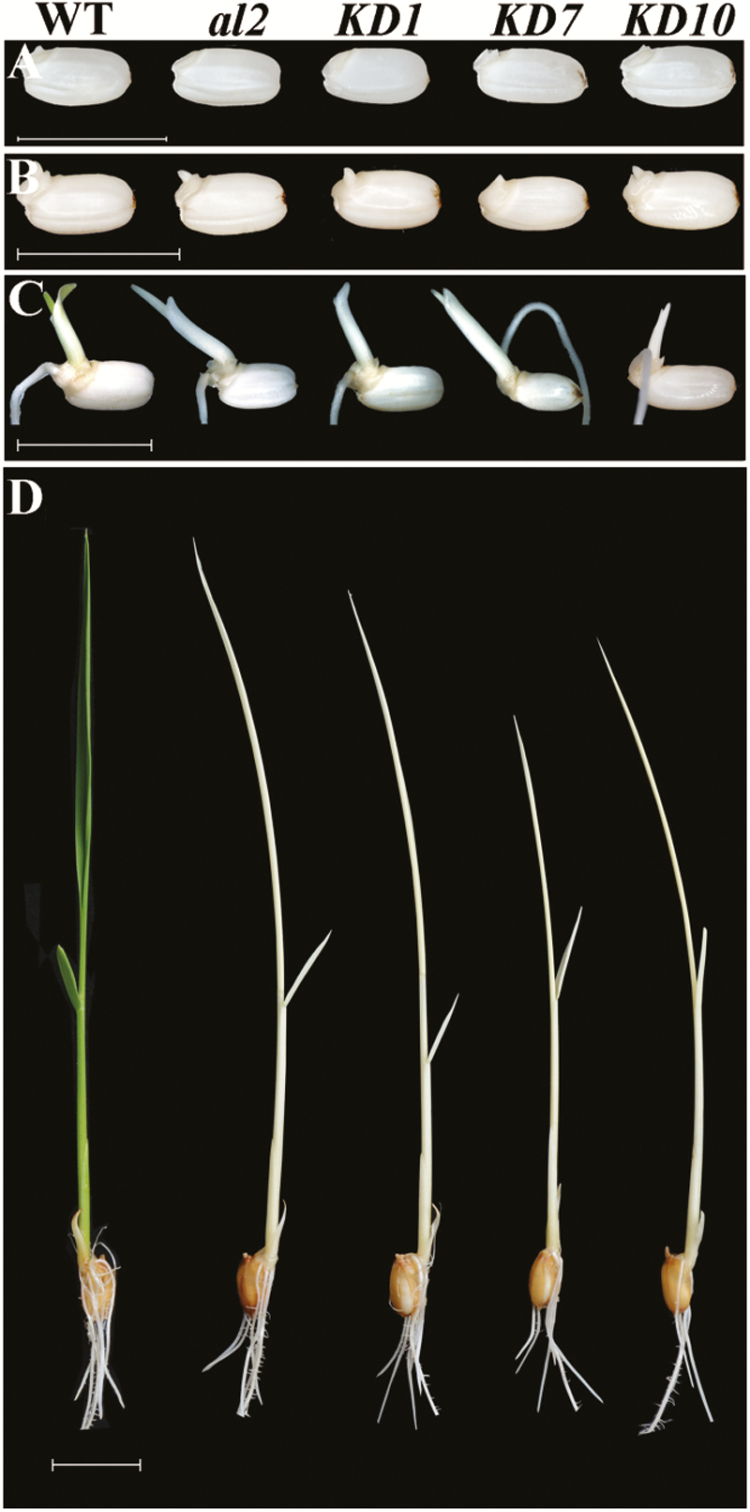
Knockdown of *AL2* leads to *al2*-like phenotype. Phenotypic analysis of the *AL2* knockdown lines (*KD*) at (A) 1 d after germination (DAG), (B) 2 DAG, (C) 4 DAG and (D) the third-leaf stage. From left to right is wild type, *al2*, *KD1*, *KD7* and *KD10*, respectively. Bar, 2cm.

The *al2* mutants were seedling lethal, which caused difficulty in the further study of the *AL2* functions. To address this issue, we produced weak *al2* alleles by specific knockdown of *AL2* gene expression. Finally, six independent knockdown lines (*KD*) were obtained with different expression levels of the *AL2* gene (Supplementary Fig. S3). In accordance with their relevant expression levels of *AL2*, the *KD* plants exhibited different degrees of the colourless phenotype. Of these 11 *KD* lines, KD1, *KD7* and *KD10*, with the most significant down-regulation of *AL2*, showed a highly similar phenotype to that of the *al2* mutants (Fig. 4A–D). These results further confirmed that *AL2* is required for chloroplast development.

### Expression pattern of *AL2*

**Fig. 5.**
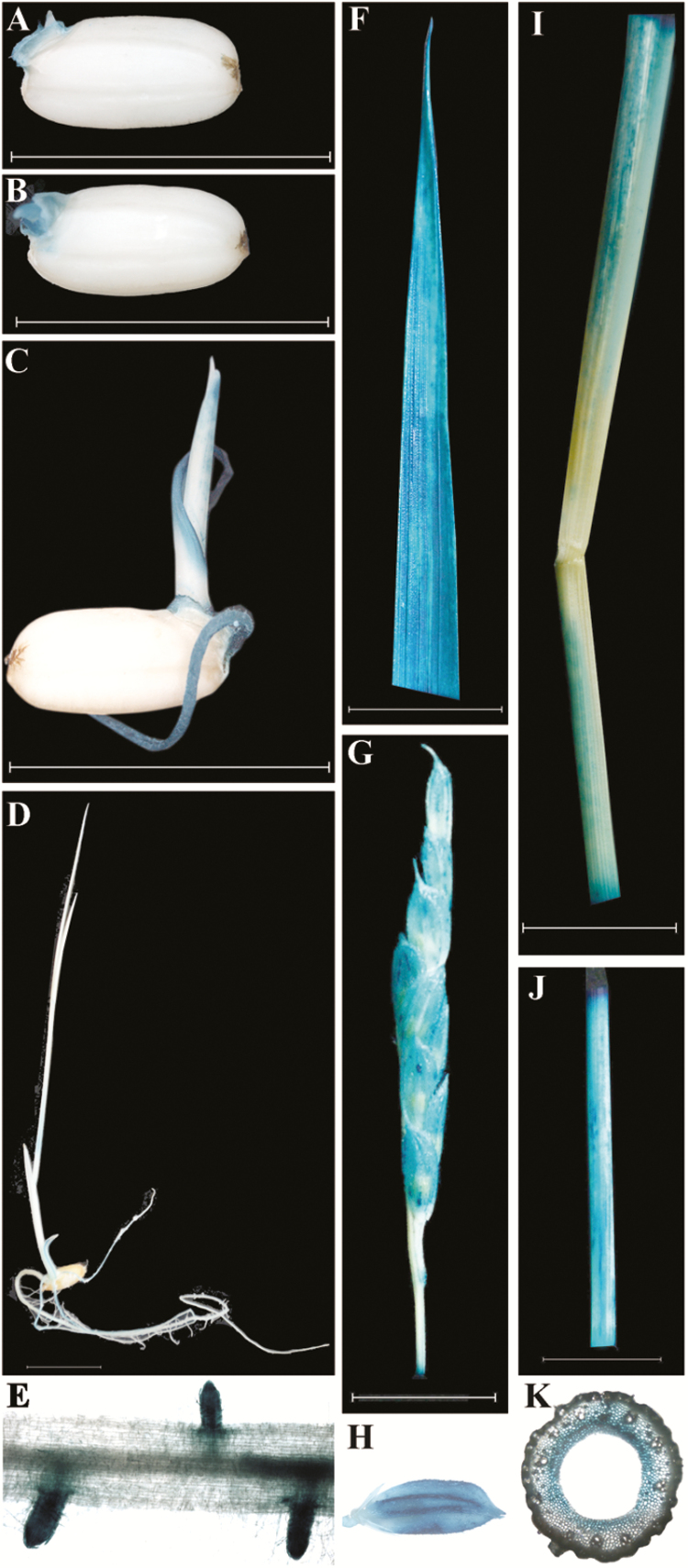
Expression pattern of *AL2*. GUS staining indicates that *AL2* is expressed at (A) 1 d after germination (DAG), (B) 2 DAG, (C) 4 DAG and (D) 10 DAG. Expression pattern of *AL2* in the (E) root, (F) leaf blade, (G) panicle, (H) spikelet, (I) leaf sheath, (J) culm and (K) endodermis of culm.

To explore the expression pattern of the *AL2* gene, its promoter was fused with the GUS reporter and transformed into wild-type plants. Consistent with the appearance of the *al2* phenotype, GUS staining revealed that the *AL2* gene was expressed during germination (Fig. 5A–D). Because plastids are also present in the root and root hairs, GUS staining was also observed in the root and root hairs in addition to the chloroplasts (Fig. 5E). Subsequently, the *AL2* gene was highly expressed in the mature leaf (Fig. 5F) and also detected in the panicle, spikelet, culm and leaf sheath (Fig. 5G–J). Histological analysis indicated that *AL2* was specifically expressed in the endodermis of the culm (Fig. 5K). Additionally, a qRT-PCR analysis of the *AL2* gene expression in various tissues revealed the similar expression pattern observed in the promoter analyses (Supplementary Fig. S4). Taken together, our results demonstrate that *AL2* is constitutively expressed in various tissues and mainly functions in the green tissues.

### *AL2* is likely to be involved in the splicing of chloroplast group I and II introns

**Fig. 6.**
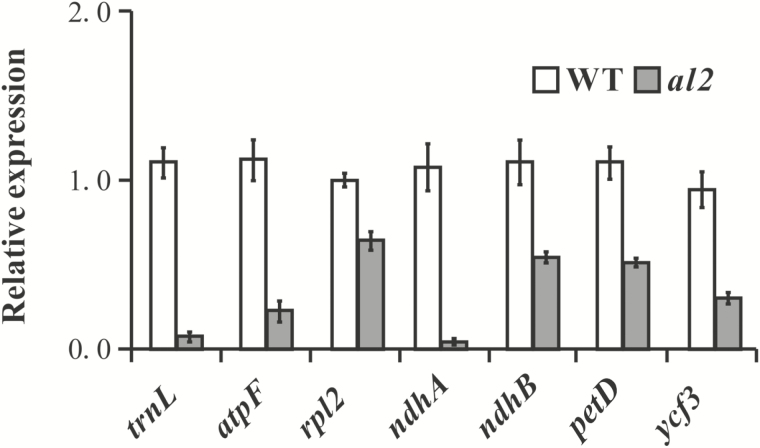
Relative expression of chloroplast genes that contain group I and II introns in *al2*. Relative expression of *trnL* (containing group I introns), and *atpF* and *rpl2* (containing group IIA introns), and *ndhA*, *ndhB*, *petD* and *ycf3* (containing group IIB introns). The values are the mean of three biological repeats with SD.

According to the gene annotation (http://rice.plantbiology.msu.edu), *AL2* encodes a putative chloroplast group IIA intron splicing facilitator CRS1 in rice. It has been implicated that *CRS1* is required for the splicing of the group IIA *atpF* intron via direct interaction (Till *et al.*, 2001). To validate the function of *AL2*, we detected the expression level of chloroplast group IIA *atpF* and *rpl2*. Our results indicated that the expression level of *atpF* and *rpl2* was significantly reduced in *al2* compared with that in wild type, suggesting that *AL2* is a functional *CRS1*. We also analysed the expression levels of chloroplast-associated genes containing group IIB introns, including *ndhA*, *ndhB*, *petD* and *ycf3*, and found that the expression of these genes was also significantly reduced in *al2* (Fig. 6), suggesting that *AL2* may also be involved in the splicing of chloroplast group IIB introns. We further tested the expression level of *trnL* containing the chloroplast group I introns. Interestingly, *trnL* expression was also predominately eliminated in the *al2* mutant (Fig. 6). Taking these results together, we propose that *AL2* is likely to be involved in the splicing of both chloroplast group I and II introns.

### Altered expression of chloroplast-associated genes in the *al2* mutants

**Fig. 7.**
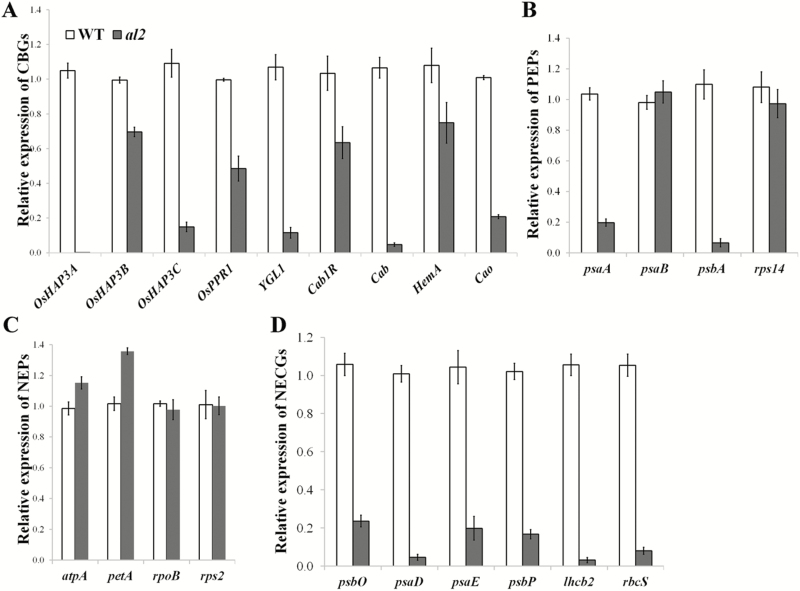
Relative expression of chloroplast-associated genes in *al2*. Relative expression of the chloroplast-associated genes in wild type and *al1*, including (A) chlorophyll biosynthetic genes (CBGs), (B) plastid-encoded polymerases (PEPs), (C) nucleus-encoded polymerases (NEPs) and (D) nuclear-encoded chloroplast genes (NECGs). Values are the mean of three biological repeats with SD.

To further determine the role of *AL2* in the biological process of chloroplast development, multiple chloroplast-associated genes were investigated. Our results indicated that the expression levels of chlorophyll biosynthetic genes (CBGs) were attenuated in the *al2* mutant (Fig. 7A). Two types of genes, PEP and NEP, play a pivotal role in chloroplast biogenesis and development, (Hedtke *et al.*, 1997; Yu *et al.*, 2014). In the *al2* mutant, the expression levels of two PEP genes were down-regulated whereas that of the other two were not significantly changed (Fig. 7B). In contrast, all four of the detected NEP genes expressed normally in *al2* compared to that in wild type (Fig. 7C), implying that *AL2*-mediated chloroplast development is independent of NEPs. Nevertheless, the transcript levels of the nuclear-encoded chloroplast genes were significantly impaired in the *al2* mutants (Fig. 7D). Taking these results together, we proposed that *AL2* coordinates the expression of a subset of chloroplast-associated genes to regulate chloroplast development in rice.

## Discussion

RNA splicing is a modification process of the nascent precursor messenger RNA transcript in which the introns are removed and exons are joined. In plants, certain chloroplast and mitochondrial genes, encoding either tRNAs or proteins, are interrupted by introns. Based on their conservation of structure and different splicing mechanisms, these introns are divided into group I and group II introns (de Longevialle *et al.*, 2010). We characterized the function of the *AL2* gene encoding CRS1 in rice. Our results suggested that *AL2* is likely to be involved in the splicing of both chloroplast group I and II introns, and it coordinates a subset of chloroplast-associated genes to regulate the development of chloroplast in rice.

The maize *CRS1* contains three CRM domains and is required for the splicing of group IIA *atpF* intron via direct interaction (Ochman *et al.*, 1988; Till *et al.*, 2001;  Ostheimer *et al.*, 2003). AL2 also contains three typical CRM domains. Moreover, multiple alignments of AL2-like proteins among various species indicated that AL2 protein is highly conserved in evolution. The albino leaf phenotype is found not only in the rice *al2* mutant but also in the Arabidopsis *crs1* mutant (Asakura and Barkan, 2006), suggesting that AL2 may be a functional CRS1 in rice. Validation of the expression of *atpF* suggested that *AL2* is involved in regulating the splicing of chloroplast group IIA introns. We thus propose that AL2 is a functional CRS1 in rice. Surprisingly, our results also showed that the expression of *ndhA*, *ndhB*, *petD*, *ycf3* and *trnL*, was significantly reduced in *al2*, suggesting that *AL2* probably participates in the splicing of group IIB and I introns. Therefore, we also propose that *AL2* not only has an overlapping role as the Arabidopsis *CRS1* but also functions distinctly. However, further studies are still required to understand the function of AL2 in the splicing of group I and IIB introns. In addition, two additional orthologous *CRS1* are present in rice, including *Os08g27150* and *Os05g47850* (de Longevialle *et al.*, 2010). Characterization of these two genes would extend our knowledge about the function of *CRS1* in chloroplast development in rice.

Phylogenetic analysis revealed that AL2-like proteins in the plant kingdom were grouped into two clusters – monocots and dicots – implying that AL2 may also have distinct roles across these plant groups. However, studies of *CRS1* in the two representative monocot and dicot plants, maize and Arabidopsis, have implicated both maize and Arabidopsis *CRS1* in the specific function of splicing of the chloroplast group II intron (Ostheimer *et al.*, 2003; Ostersetzer *et al.*, 2005; Asakura and Barkan, 2006). Therefore, we propose that the role of *AL2* in intron splicing in rice may be different from the above plants. This assumption would be an interesting issue to explore in the future. So far, numerous genes have been shown to be crucial for chloroplast biogenesis and development, and mutations of these genes cause chlorotic or bleached leaf phenotypes. Similar to *al2*, *snow-white leaf1* (*swl1*) and *albino lethal 1* (*al1*) also exhibit albino leaf and seedling lethality phenotype in rice (Hayashi-Tsugane *et al.*, 2014; Zhao *et al.*, 2016), whereas other mutants, such as *sco* and *var*, generate a leaf phenotype restricted to one leaf organ in Arabidopsis. Therefore, characterization of many more monocot mutants will facilitate the discovery of genes functioning in chloroplast biogenesis and/or development that have not yet been clarified in dicot research.

The most abundant transcript of *AL2* is found in green tissues, such as the leaf and culm. However, *AL2* is also expressed in non-green tissues, such as the root. This suggests a possible involvement of *AL2* in the regulation of RNA processing in different kinds of plastids in addition to the chloroplast. However, this hypothesis requires further determination as it remains unclear whether chloroplast intron splicing factors participate in the regulation of other chloroplast-associated genes. In this study, we showed that *AL2* is also involved in the regulation of PEPs and nuclear-encoded chloroplast genes. By contrast, our results indicate that *AL2* is not involved in the regulation of the NEPs, suggesting that *AL2*-mediated chloroplast development is independent of NEPs. Additionally, we found that a group of CBGs was significantly down-regulated in the *al2* mutants, which may be due to the absence of apoproteins for chlorophyll rather than to the direct involvement of *AL2* in regulating these CBGs.

Overall, our results showed that *AL2* is likely to be involved in the splicing of both group I and II introns in rice, which is distinct from the function of orthologous CRS1 in maize and Arabidopsis. Moreover, our results also indicated that *AL2* coordinates a subset of chloroplast-associated genes to regulate the development of chloroplast in rice.

## Supplementary data

Supplementary data are available at *JXB* online.

Figure S1. Phylogenetic tree of AL2-like proteins among multiple organisms.

Figure S2. Chlorophyll contents of complementation lines.

Figure S3. Expression pattern of the *AL2* gene in the *AL2* knock-down lines.

Figure S4. Expression pattern of the *AL2* gene in various tissues.

Table S1. Primers used in this study.

Supplementary Data

## References

[CIT0001] Albrecht V Ingenfeld A Apel K 2006 Characterization of the snowy cotyledon 1 mutant of Arabidopsis thaliana: the impact of chloroplast elongation factor G on chloroplast development and plant vitality. Plant Molecular Biology 60, 507–518. 16525888 10.1007/s11103-005-4921-0

[CIT0002] Albrecht V Ingenfeld A Apel K 2008 Snowy cotyledon 2: the identification of a zinc finger domain protein essential for chloroplast development in cotyledons but not in true leaves. Plant Molecular Biology 66, 599–608. 18209955 10.1007/s11103-008-9291-y

[CIT0003] Albrecht V Simkova K Carrie C Delannoy E Giraud E Whelan J Small ID Apel K Badger MR Pogson BJ 2010 The cytoskeleton and the peroxisomal-targeted snowy cotyledon3 protein are required for chloroplast development in Arabidopsis. The Plant Cell 22, 3423–3438. 20978221 10.1105/tpc.110.074781 PMC2990128

[CIT0004] Asakura Y Barkan A 2006 Arabidopsis orthologs of maize chloroplast splicing factors promote splicing of orthologous and species-specific group II introns. Plant Physiology 142, 1656–1663. 17071648 10.1104/pp.106.088096 PMC1676066

[CIT0005] Asakura Y Barkan A 2007 A CRM domain protein functions dually in group I and group II intron splicing in land plant chloroplasts. The Plant Cell 19, 3864–3875. 18065687 10.1105/tpc.107.055160 PMC2217638

[CIT0006] Asakura Y Bayraktar OA Barkan A 2008 Two CRM protein subfamilies cooperate in the splicing of group IIB introns in chloroplasts. RNA 14, 2319–2332. 18799595 10.1261/rna.1223708 PMC2578863

[CIT0007] Barkan A Klipcan L Ostersetzer O Kawamura T Asakura Y Watkins KP 2007 The CRM domain: an RNA binding module derived from an ancient ribosome-associated protein. RNA 13, 55–64. 17105995 10.1261/rna.139607 PMC1705760

[CIT0008] Bonen L Vogel J 2001 The ins and outs of group II introns. Trends in Genetics 17, 322–331. 11377794 10.1016/s0168-9525(01)02324-1

[CIT0009] Borner T Aleynikova AY Zubo YO Kusnetsov VV 2015 Chloroplast RNA polymerases: Role in chloroplast biogenesis. Biochimica et Biophysica Acta 1847, 761–769. 25680513 10.1016/j.bbabio.2015.02.004

[CIT0010] Chen Q Xie Q Gao J 2015 Characterization of Rolled and Erect Leaf 1 in regulating leave morphology in rice. Journal of Experimental Botany 66, 6047–6058. 26142419 10.1093/jxb/erv319 PMC4566990

[CIT0011] de Longevialle AF Meyer EH Andres C Taylor NL Lurin C Millar AH Small ID 2007 The pentatricopeptide repeat gene OTP43 is required for trans-splicing of the mitochondrial nad1 Intron 1 in *Arabidopsis thaliana* . The Plant Cell 19, 3256–3265. 17965268 10.1105/tpc.107.054841 PMC2174710

[CIT0012] de Longevialle AF Small ID Lurin C 2010 Nuclearly encoded splicing factors implicated in RNA splicing in higher plant organelles. Molecular Plant 3, 691–705. 20603383 10.1093/mp/ssq025

[CIT0013] Hammani K Barkan A 2014 An mTERF domain protein functions in group II intron splicing in maize chloroplasts. Nucleic Acids Research 42, 5033–5042. 24500208 10.1093/nar/gku112 PMC4005652

[CIT0014] Hartmut K 1983 Determinations of total carotenoids and chlorophylls b of leaf extracts in different solvents. Analysis 4, 142–196.

[CIT0015] Hayashi-Tsugane M Takahara H Ahmed N Himi E Takagi K Iida S Tsugane K Maekawa M 2014 A mutable albino allele in rice reveals that formation of thylakoid membranes requires the SNOW-WHITE LEAF1 gene. Plant & Cell Physiology 55, 3–15. 24151203 10.1093/pcp/pct149

[CIT0016] Hedtke B Borner T Weihe A 1997 Mitochondrial and chloroplast phage-type RNA polymerases in Arabidopsis. Science 277, 809–811. 9242608 10.1126/science.277.5327.809

[CIT0017] Jarvis P Lopez-Juez E 2013 Biogenesis and homeostasis of chloroplasts and other plastids. Nature Reviews Molecular Cell Biology 14, 787–802. 24263360 10.1038/nrm3702

[CIT0018] Jefferson RA Kavanagh TA Bevan MW 1987 GUS fusions: beta-glucuronidase as a sensitive and versatile gene fusion marker in higher plants. The EMBO Journal 6, 3901–3907. 3327686 10.1002/j.1460-2075.1987.tb02730.x PMC553867

[CIT0019] Jenkins BD Kulhanek DJ Barkan A 1997 Nuclear mutations that block group II RNA splicing in maize chloroplasts reveal several intron classes with distinct requirements for splicing factors. The Plant Cell 9, 283–296. 9090875 10.1105/tpc.9.3.283 PMC156918

[CIT0020] Keren I Bezawork-Geleta A Kolton M Maayan I Belausov E Levy M Mett A Gidoni D Shaya F Ostersetzer-Biran O 2009 AtnMat2, a nuclear-encoded maturase required for splicing of group-II introns in Arabidopsis mitochondria. RNA 15, 2299–2311. 19946041 10.1261/rna.1776409 PMC2779688

[CIT0021] Keren I Tal L des Francs-Small CC Araujo WL Shevtsov S Shaya F Fernie AR Small I Ostersetzer-Biran O 2012 nMAT1, a nuclear-encoded maturase involved in the trans-splicing of nad1 intron 1, is essential for mitochondrial complex I assembly and function. Plant Journal 71, 413–426. 22429648 10.1111/j.1365-313X.2012.04998.x

[CIT0022] Kessler F Schnell D 2009 Chloroplast biogenesis: diversity and regulation of the protein import apparatus. Current Opinion in Cell Biology 21, 494–500. 19410443 10.1016/j.ceb.2009.03.004

[CIT0023] Kroeger TS Watkins KP Friso G van Wijk KJ Barkan A 2009 A plant-specific RNA-binding domain revealed through analysis of chloroplast group II intron splicing. Proceedings of the National Academy of Sciences, USA 106, 4537–4542. 10.1073/pnas.0812503106 PMC2657376 19251672

[CIT0024] Kruger K Grabowski PJ Zaug AJ Sands J Gottschling DE Cech TR 1982 Self-splicing RNA: autoexcision and autocyclization of the ribosomal RNA intervening sequence of Tetrahymena. Cell 31, 147–157. 6297745 10.1016/0092-8674(82)90414-7

[CIT0025] Kuhn K Carrie C Giraud E Wang Y Meyer EH Narsai R des Francs-Small CC Zhang B Murcha MW Whelan J 2011 The RCC1 family protein RUG3 is required for splicing of nad2 and complex I biogenesis in mitochondria of Arabidopsis thaliana. Plant Journal 67, 1067–1080. 21623974 10.1111/j.1365-313X.2011.04658.x

[CIT0026] Liu X Yu F Rodermel S 2010 Arabidopsis chloroplast FtsH, var2 and suppressors of var2 leaf variegation: a review. Journal of Integrative Plant Biology 52, 750–761. 20666930 10.1111/j.1744-7909.2010.00980.x

[CIT0027] Liu Y He J Chen Z Ren X Hong X Gong Z 2010 ABA overly-sensitive 5 (ABO5), encoding a pentatricopeptide repeat protein required for cis-splicing of mitochondrial nad2 intron 3, is involved in the abscisic acid response in Arabidopsis. Plant Journal 63, 749–765. 20561255 10.1111/j.1365-313X.2010.04280.x

[CIT0028] Lopez-Juez E Pyke KA 2005 Plastids unleashed: their development and their integration in plant development. The International Journal of Developmental Biology 49, 557–577. 16096965 10.1387/ijdb.051997el

[CIT0029] Michel F Umesono K Ozeki H 1989 Comparative and functional anatomy of group II catalytic introns – a review. Gene 82, 5–30. 2684776 10.1016/0378-1119(89)90026-7

[CIT0030] Ochman H Gerber AS Hartl DL 1988 Genetic applications of an inverse polymerase chain reaction. Genetics 120, 621–623. 2852134 10.1093/genetics/120.3.621 PMC1203539

[CIT0031] Ostersetzer O Cooke AM Watkins KP Barkan A 2005 CRS1, a chloroplast group II intron splicing factor, promotes intron folding through specific interactions with two intron domains. The Plant Cell 17, 241–255. 15598799 10.1105/tpc.104.027516 PMC544502

[CIT0032] Ostheimer GJ Williams-Carrier R Belcher S Osborne E Gierke J Barkan A 2003 Group II intron splicing factors derived by diversification of an ancient RNA-binding domain. The EMBO Journal 22, 3919–3929. 12881426 10.1093/emboj/cdg372 PMC169045

[CIT0033] Pogson BJ Albrecht V 2011 Genetic dissection of chloroplast biogenesis and development: an overview. Plant Physiology 155, 1545–1551. 21330494 10.1104/pp.110.170365 PMC3091115

[CIT0034] Sakamoto W Miyagishima SY Jarvis P 2008 Chloroplast biogenesis: control of plastid development, protein import, division and inheritance. The Arabidopsis Book 6, e0110. 22303235 10.1199/tab.0110 PMC3243408

[CIT0035] Till B Schmitz-Linneweber C Williams-Carrier R Barkan A 2001 CRS1 is a novel group II intron splicing factor that was derived from a domain of ancient origin. RNA 7, 1227–1238. 11565746 10.1017/s1355838201010445 PMC1370168

[CIT0036] Toki S Hara N Ono K Onodera H Tagiri A Oka S Tanaka H 2006 Early infection of scutellum tissue with Agrobacterium allows high-speed transformation of rice. Plant Journal 47, 969–976. 16961734 10.1111/j.1365-313X.2006.02836.x

[CIT0037] Toor N Hausner G Zimmerly S 2001 Coevolution of group II intron RNA structures with their intron-encoded reverse transcriptases. RNA 7, 1142–1152. 11497432 10.1017/s1355838201010251 PMC1370161

[CIT0038] Watkins KP Kroeger TS Cooke AM Williams-Carrier RE Friso G Belcher SE van Wijk KJ Barkan A 2007 A ribonuclease III domain protein functions in group II intron splicing in maize chloroplasts. The Plant Cell 19, 2606–2623. 17693527 10.1105/tpc.107.053736 PMC2002627

[CIT0039] Yu QB Huang C Yang ZN 2014 Nuclear-encoded factors associated with the chloroplast transcription machinery of higher plants. Frontiers in Plant Science 5, 316. 25071799 10.3389/fpls.2014.00316 PMC4080259

[CIT0040] Zhao DS Zhang CQ Li QF Yang QQ Gu MH Liu QQ 2016 A residue substitution in the plastid ribosomal protein L12/AL1 produces defective plastid ribosome and causes early seedling lethality in rice. Plant Molecular Biology 91, 161–177. 26873698 10.1007/s11103-016-0453-z

[CIT0041] Zmudjak M Colas des Francs-Small C Keren I Shaya F Belausov E Small I Ostersetzer-Biran O 2013 mCSF1, a nucleus-encoded CRM protein required for the processing of many mitochondrial introns, is involved in the biogenesis of respiratory complexes I and IV in Arabidopsis. New Phytologist 199, 379–394. 23646912 10.1111/nph.12282

[CIT0042] Zoschke R Nakamura M Liere K Sugiura M Borner T Schmitz-Linneweber C 2010 An organellar maturase associates with multiple group II introns. Proceedings of the National Academy of Sciences, USA 107, 3245–3250. 10.1073/pnas.0909400107 PMC2840290 20133623

