# Supplementary material for: Albino Leaf 2 is involved in the splicing of chloroplast group I and II introns in rice
Source: J Exp Bot. 2016 Aug 19;67(18):5339–47. doi: 10.1093/jxb/erw296 (PMC5049385; doi:10.1093/jxb/erw296)
Supplement: Supplementary Data [file supp_67_18_5339__index.html]

 Albino Leaf 2 is involved in the splicing of chloroplast group I and II introns in rice — Albino Leaf 2 is involved in the splicing of chloroplast group I and II introns in rice — Supplementary Data 

# *Albino Leaf 2* is involved in the splicing of chloroplast group I and II introns in rice

## Supplementary Data

Data files

- supplementary\_figures\_S1\_S4\_table\_S1.pdf - Supplementary Data
