# Supplementary material for: Albino Leaf 2 is involved in the splicing of chloroplast group I and II introns in rice
Source: J Exp Bot. 2016 Aug 19;67(18):5339–47. doi: 10.1093/jxb/erw296 (PMC5049385; doi:10.1093/jxb/erw296)
Supplement: Supplementary Data [file supp_erw296_supplementary_figures_S1_S4_table_S1.pdf]

Supplemental Table S1. Primers used in this study.

	Name	Sequence of Primers (5'-3')	Annotation/vector
Flanking of T-DNA	C1	5'-TggCgTAATAgCgAAgAggCC-3'	T-DNA right border primer#1 for inverse PCR
	H1	5'-AATAACAgAgTCTAgCACCTCg-3'	T-DNA left border primer#2 for inverse PCR
	C2	5'-AATggCgAATgCTAgAgC-3'	T-DNA right border primer#3 for inverse PCR
	H2	5'-CTACCCAATCTTTTgTgC-3'	T-DNA left border primer#4 for inverse PCR
	F1	5'-ACCAgATgCggAgTTACTTCA-3'	flanking of T-DNA
	R1	5'-TCCTCAggTTCCTCTTCATCC-3'	with 5TF1 for test flanking of T-DNA
	5TR1	5'-TCgTCCgAgggCAAAgAAATAgA-3'	T-DNA right border
	5TF1	5'-gCACCgATCgCCCTTCCCAACA-3'	T-DNA left border
Antisense	anti-f	5'-ggCCggATCCCAATTggTTTCTgACgAgg-3'	P130135Snos
	anti-r	5'-ggCCgTCgACCCACAATgAgCTgAgTgACCC-3'	
Promoter	pro-f	5'-TTgCATgCCTgCAgggTgggAATTCgCTTggAT-3′	P1300GUSnos
	pro-r	5'-ggggatcctctagaggAgAggAgCTCCgATgAgAgA-3′	
Complementation	Comp-f	5'-ggCCAAgCTTgTgATTTTgAACCCggTTTTATCT-3'	pCAMBIA 1301
	Comp-r	5'-ggCCggTACCAATCgCgCTCTCAgCTTTgA-3'	
qRT-PCR	410q-f1	5'-CCATgTTTCTTCAggTggTg-3'	
	410q-r1	5'-CACTCAATCgCAgCATTTCT-3'	
	410q-f2	5'-ggACTgAATCgCTCAgCATA-3'	
	410q-r2	5'-AAgCAAgTCAgCATCCACAg-3'	
	OsHAP3A-f	5'-TCTgTTAAggAAgAACCCAC-3'	
	OsHAP3A-r	5'-TAgATTTgTgCCACCTgATA-3'	
	OsHAP3B-f	5'-AACTgCAAAggCTggTgATggCTCT-3'	
	OsHAP3B-r	5'-TACATCTgAgAAgCAgCCTTggCTC-3'	
	OsHAP3C-f	5'-ggTCAATgggCACgCTCggATTCg-3'	
	OsHAP3C-r	5'-ggAACTTTAgAAgCATCCTgCTTAC-3'	
	OsPPR1-f	5'-CATTgATTTCgACgTACACTA-3'	
	OsPPR1-r	5'-AgCATTTgTCATTCggTCT-3'	
	YGL1-f	5'-TCTTggTgCgAgCTACATTg-3'	
	YGL1-r	5'-gCTTgCCTgAACTgAAAAgg-3'	
	cab1R-f	5'-AgATgggTTTAgTgCgACgAg-3'	
	cab1R-r	5'-TTTgggATCgAgggAgTATTT-3'	
	HEMA1-f	5'-CgCTATTTCTgATgCTATgggT-3'	
	HEMA1-r	5'-TCTTgggTgATgATTgTTTgg-3'	
	Cao-f	5'-TCAACCATTggCATCTCAAA-3'	
	Cao-r	5'-CgTgATgCTgTCgCTAgTgT-3'	
	psaA-f	5'-TTAgAAATCCgCCAATCCA-3'	
	psaA-r	5'-TgCTAggCTCTACAACCATT-3'	
	psaB-f	5'-gAgCAATATCggTCAgCCACA-3'	
	psaB-r	5'-ACCACTCAAggAgCgggAAC-3'	
	psbA-f	5'-ACCCTCATTAgCAgATTCgT-3'	
	psbA-r	5'-gATTgTATTCCAggCAgAgC-3'	
	rps14-f	5'-TCACTCAAACTCAAAgggTA-3'	
	rps14-r	5'-AAgCggCAgAAATTAgAAC-3'	
	atpA-f	5'-TATCggTCAAAgAgCATC-3'	
	atpA-r	5'-CgTATAAggAgCgAggTA-3'	
	petA-f	5'-TgCCATTTAgCgAATAAgCC-3'	
	petA-r	5'-CCACATTCAACCCTCCCTTT-3'	
	rpoB-f	5'-TggTACATATCCCTTATCTCAA-3'	
	rpoB-r	5'-CTCCAggACCCAAACAACTC-3'	
	rps2-f	5'-gAgATgATAgAAgCgggAgTT-3'	
	rps2-r	5'-TAACATAATgACAACgAgCC-3'	
	psbO-f	5'-gCTCTACCggCTACgACAAC-3'	
	psbO-r	5'-TgACATCCTTgggCACCTT-3'	
	psaD-f	5'-CCgCTCCAAgTACAAgATCA-3'	
	psaD-r	5'-AAgAgCAgCCTgACAgATgA-3'	
	psaE-f	5'-CCgCCAAgCCgCCTCCCATT-3'	
	psaE-r	5'-AgCTCgACgACgATCCATCC-3'	
	psbP-f	5'-AAgACAgATTCCgAgggTgg-3'	
	psbP-r	5'-TgATTCgCTAgggATTAAAgAg-3'	
	Lhcb2-f	5'-CCCCATCgAgAACCTCTTC-3'	
	Lhcb2-r	5'-CggTgCgTggCTACTACAA-3'	

	rcbs-f	5'-TgAgggCATCAAgAAgTT-3’	
	rcbs-r	5'-CgATgATACggACAAAgg-3’	
	actin-f	5'-AggAAggCTggAAgAggACC-3’	
	actin-r	5'-CgggAAATTgTgAgggACAT-3’	
	atpF-F1	5'-ggAgTgTgTgCgAgTTgTCT-3’	
	atpF-R1	5'-TTTCgAgCTgCTCAATggT-3’	
	atpF-R2	5'-TTCCAAACTAATAgAAgTggCATT-3’	
	trnL-F1	5'-ATCCTgAgCCAAATCCATgT-3’	
	trnL-R1	5'-gAgTTCCgCTAgCAACACAA-3’	
	ndhA-F1	5'-TTCTTATggCAggATATAgCTCA-3’	
	ndhA-R1	5'-TCAACTgTACTTgAACTgTTggATAA-3’	
	ndhB-F1	5'-CCCCTTTTCATCAATggACT-3’	
	ndhB-R1	5'-TgAAgCAgCAACTTTCgAAg-3’	
	rpl2-F1	5'-gggTgggCAATTAgCTAgAg-3’	
	rpl2-R1	5'-gTggCCgATTTACCTTCTTT-3’	
	rpl2-F2	5'-AggCAAgTgAAATCCAATCC-3’	
	rpl2-R2	5'-CCTCTATgCCTTgCggTAAT-3’	
	ycf3-F1	5'-gCgAATAATTCCgACAACCT-3’	
	ycf3-R1	5'-gCTTCCgCATAATTTCCTTC-3’	
	petD-F1	5'-gATCCgTTTgCAACTCCTCT-3’	
	petD-R1	5'-CCATTAAgAgAACgCCCAAT-3’	

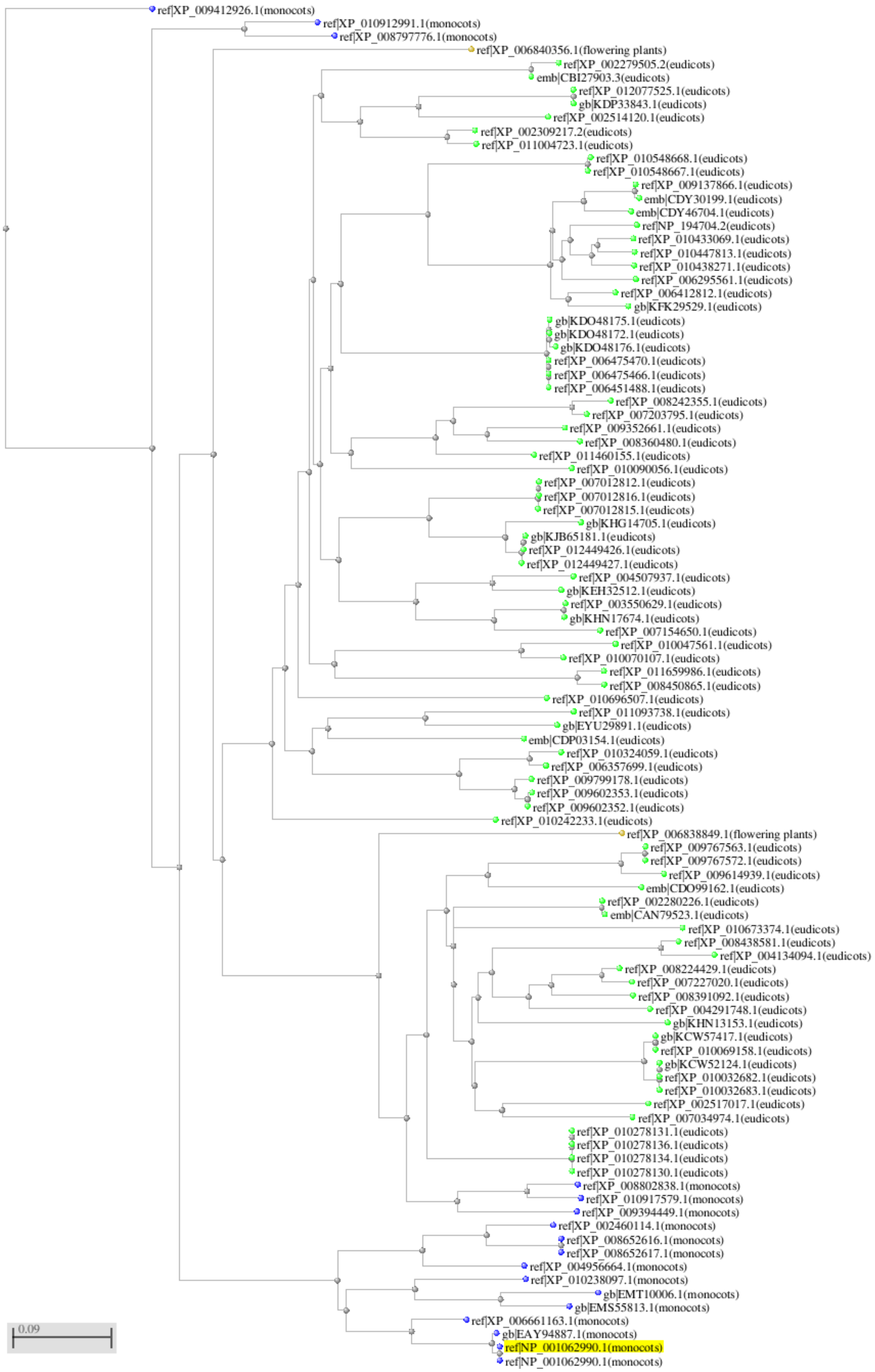
ref|XP_009412926.1(monocots)
ref|XP_010912991.1(monocots)
ref|XP_008797776.1(monocots)
ref|XP_006840356.1(flowering plants)
ref|XP_002279505.2(eudicots)
emb|CBI27903.3(eudicots)
ref|XP_012077525.1(eudicots)
gb|KDP33843.1(eudicots)
ref|XP_002514120.1(eudicots)
ref|XP_002309217.2(eudicots)
ref|XP_011004723.1(eudicots)
ref|XP_010548668.1(eudicots)
ref|XP_010548667.1(eudicots)
ref|XP_009137866.1(eudicots)
emb|CDY30199.1(eudicots)
emb|CDY46704.1(eudicots)
ref|NP_194704.2(eudicots)
ref|XP_010433069.1(eudicots)
ref|XP_010447813.1(eudicots)
ref|XP_010438271.1(eudicots)
ref|XP_006295561.1(eudicots)
ref|XP_006412812.1(eudicots)
gb|KFK29529.1(eudicots)
gb|KDO48175.1(eudicots)
gb|KDO48172.1(eudicots)
gb|KDO48176.1(eudicots)
ref|XP_006475470.1(eudicots)
ref|XP_006475466.1(eudicots)
ref|XP_006451488.1(eudicots)
ref|XP_008242355.1(eudicots)
ref|XP_007203795.1(eudicots)
ref|XP_009352661.1(eudicots)
ref|XP_008360480.1(eudicots)
ref|XP_011460155.1(eudicots)
ref|XP_010090056.1(eudicots)
ref|XP_007012812.1(eudicots)
ref|XP_007012816.1(eudicots)
ref|XP_007012815.1(eudicots)
gb|KHG14705.1(eudicots)
gb|KJB65181.1(eudicots)
ref|XP_012449426.1(eudicots)
ref|XP_012449427.1(eudicots)
ref|XP_004507937.1(eudicots)
gb|KEH32512.1(eudicots)
ref|XP_003550629.1(eudicots)
gb|KHN17674.1(eudicots)
ref|XP_007154650.1(eudicots)
ref|XP_010047561.1(eudicots)
ref|XP_010070107.1(eudicots)
ref|XP_011659986.1(eudicots)
ref|XP_008450865.1(eudicots)
ref|XP_010696507.1(eudicots)
ref|XP_011093738.1(eudicots)
gb|EYU29891.1(eudicots)
emb|CDP03154.1(eudicots)
ref|XP_010324059.1(eudicots)
ref|XP_006357699.1(eudicots)
ref|XP_009799178.1(eudicots)
ref|XP_009602353.1(eudicots)
ref|XP_009602352.1(eudicots)
ref|XP_010242233.1(eudicots)
ref|XP_006838849.1(flowering plants)
ref|XP_009767563.1(eudicots)
ref|XP_009767572.1(eudicots)
ref|XP_009614939.1(eudicots)
emb|CDO99162.1(eudicots)
ref|XP_002280226.1(eudicots)
emb|CAN79523.1(eudicots)
ref|XP_010673374.1(eudicots)
ref|XP_008438581.1(eudicots)
ref|XP_004134094.1(eudicots)
ref|XP_008224429.1(eudicots)
ref|XP_007227020.1(eudicots)
ref|XP_008391092.1(eudicots)
ref|XP_004291748.1(eudicots)
gb|KHN13153.1(eudicots)
gb|KCW57417.1(eudicots)
ref|XP_010069158.1(eudicots)
gb|KCW52124.1(eudicots)
ref|XP_010032682.1(eudicots)
ref|XP_010032683.1(eudicots)
ref|XP_002517017.1(eudicots)
ref|XP_007034974.1(eudicots)
ref|XP_010278131.1(eudicots)
ref|XP_010278136.1(eudicots)
ref|XP_010278134.1(eudicots)
ref|XP_010278130.1(eudicots)
ref|XP_008802838.1(monocots)
ref|XP_010917579.1(monocots)
ref|XP_009394449.1(monocots)
ref|XP_002460114.1(monocots)
ref|XP_008652616.1(monocots)
ref|XP_008652617.1(monocots)
ref|XP_004956664.1(monocots)
ref|XP_010238097.1(monocots)
gb|EMT10006.1(monocots)
gb|EMS55813.1(monocots)
ref|XP_006661163.1(monocots)
gb|EAY94887.1(monocots)
ref|NP_001062990.1(monocots)
ref|NP_001062990.1(monocots)
0.09

Supplemental Figure S1. Phylogenetic tree of AL2-like proteins among multiple organisms. The AL2 protein sequence (NCBI accession no: NP_001062990.1) was used to do the BLASTp, and all 100 the putative AL2-like proteins were used to generate the phylogenetic tree from the Blast Tree View. GeneBank accession numbers of each AL2 protein and species (monocot or eudicot) are listed in parentheses.

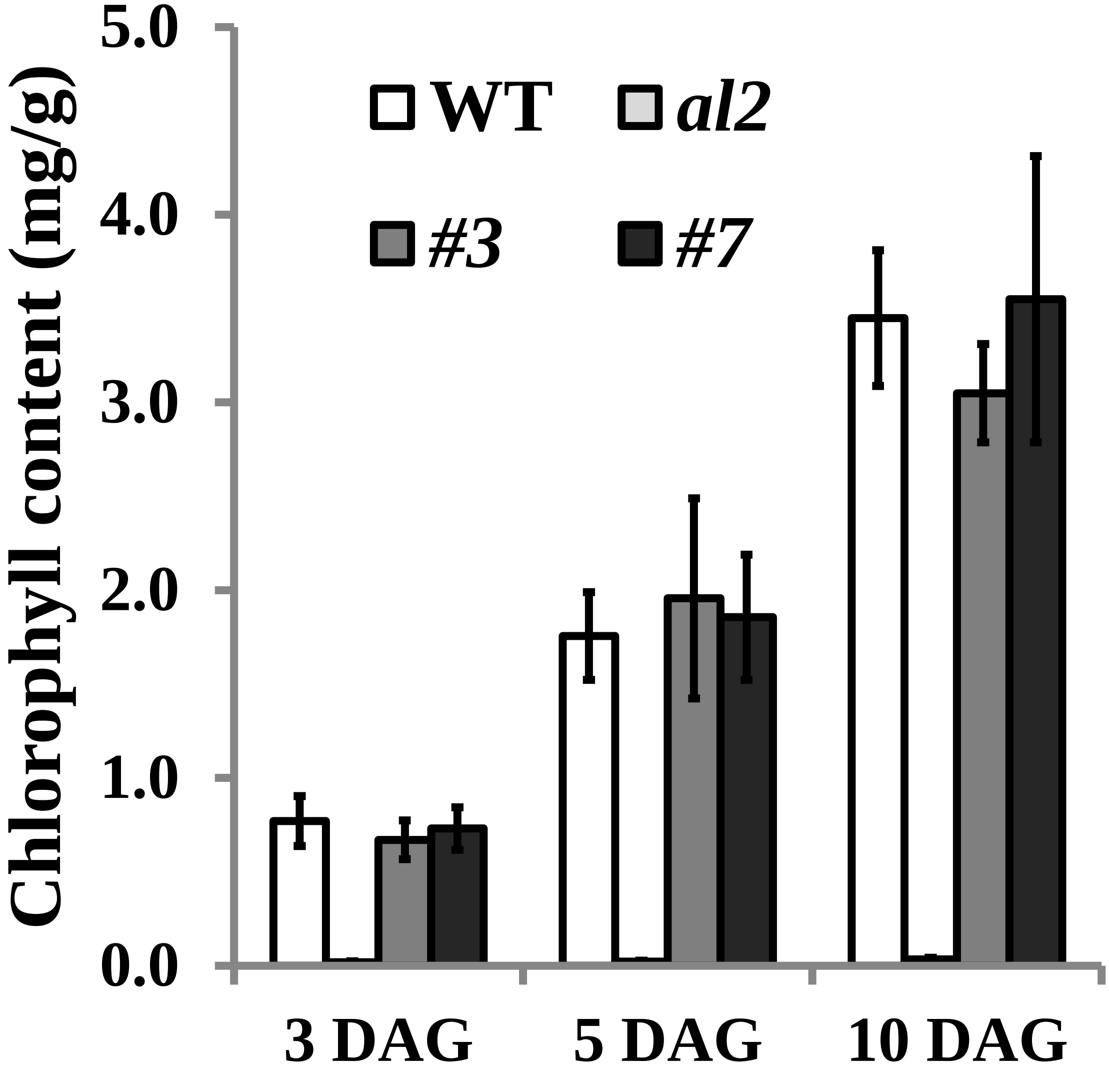

Supplemental Figure S2. Chlorophyll contents of complementation lines. #3 and #7 are the *AL2* complementing lines 3 and 7, respectively. DAG, day after germination. Values are the mean of three biological repeats.

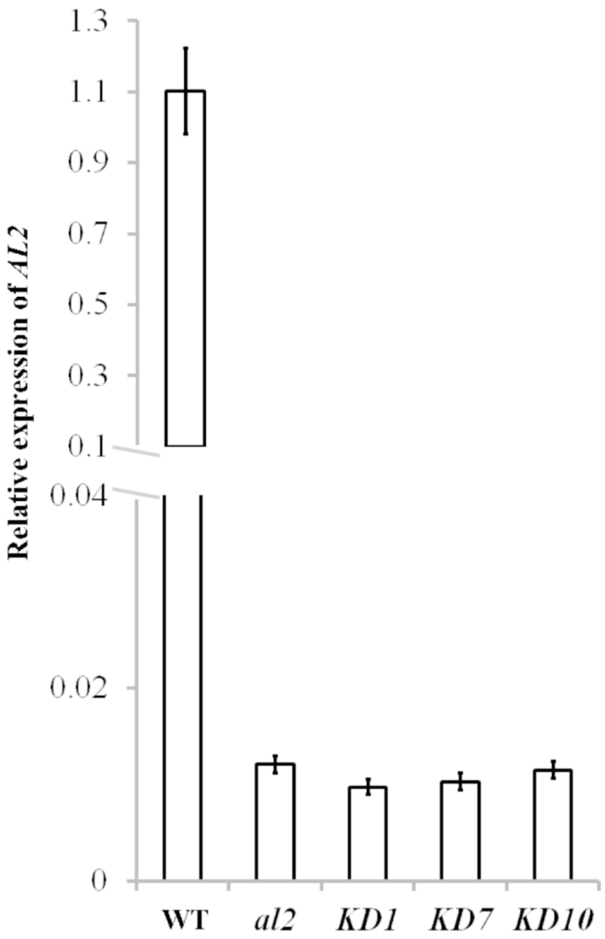

Supplemental Figure S3. Expression pattern of the *AL2* gene in the *AL2*-knock down lines. Relative expression of *AL2* in wild type, *al2*, *AL2* knockdown line 1 (*KD1*), *KD7* and *KD10* was determined by quantitative real time PCR (qRT-PCR). Values are the mean of three biological repeats with the SD.

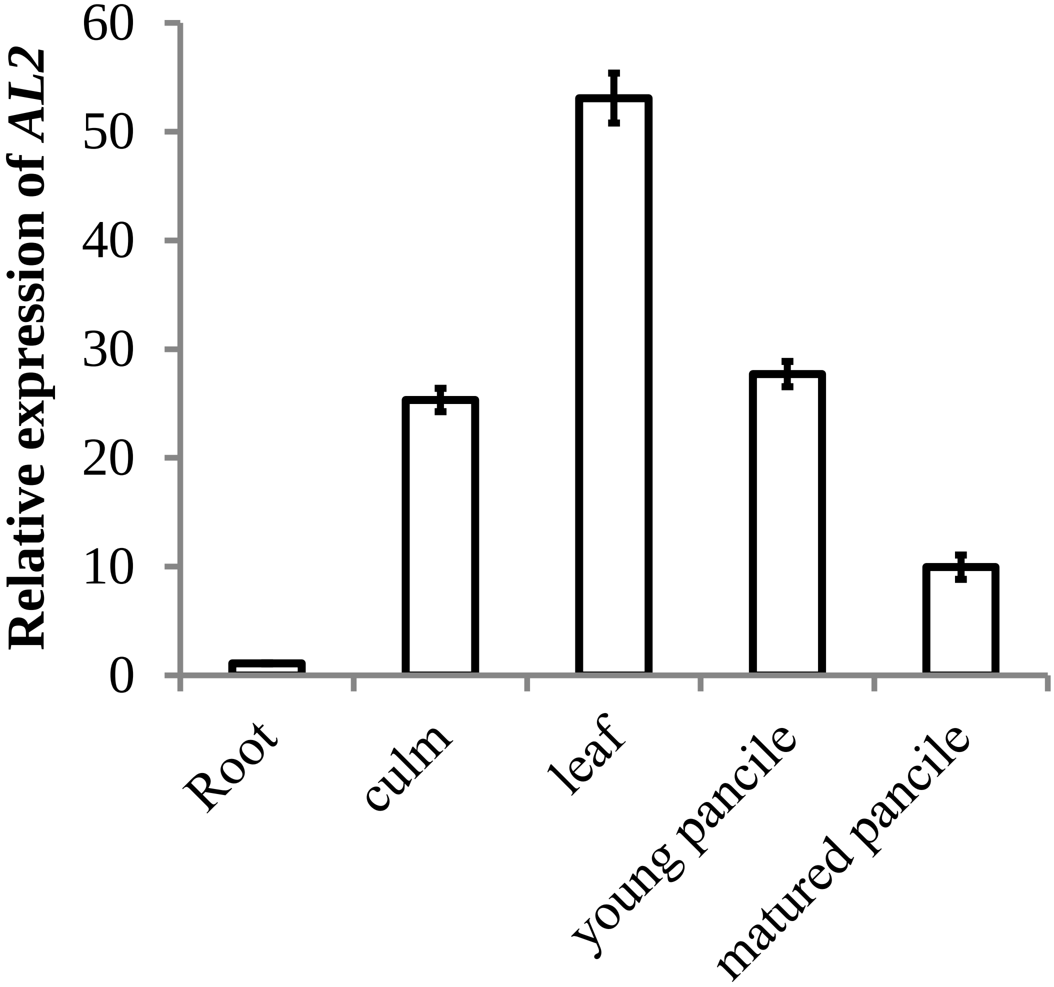

Supplemental Figure S4. Expression pattern of *AL2* gene in various tissues. Relative expression of *AL2* was determined by quantitative real time PCR (qRT-PCR). The tissues used in this experiment were derived from the heading stage. Values are the mean of three biological repeats with the SD.
